# Early Childhood Anemia in a Birth Cohort in Coastal Kenya: Links to Infection and Nutrition

**DOI:** 10.4269/ajtmh.17-0688

**Published:** 2019-05-06

**Authors:** Julia Kao, Francis Mutuku, Shanique Martin, Justin Lee, Jackson Mwandi, Dunstan Mukoko, Indu Malhotra, Charles H. King, Angelle Desiree LaBeaud

**Affiliations:** 1 Stanford University School of Medicine, Stanford, California;; 2 Technical University of Mombasa, Mombasa, Kenya;; 3 Vector Borne Disease Control Unit, Msambweni Field Laboratory, Kwale County, Kenya;; 4 Vector-Borne Diseases Control Unit, Ministry of Health, Nairobi, Kenya;; 5 Center for Global Health and Diseases, Case Western Reserve University, Cleveland, Ohio

## Abstract

Anemia is known to impact a child’s growth and development, but not all anemias are caused by iron deficiency, and the CDC and WHO have emphasized investigating other contributors to anemia. This cross-sectional sub-study of a 2012–2016 maternal-child cohort in coastal Kenya evaluated 244 children and found 185 (76%) to have been anemic on at least one time point since birth. At the time of assessment in 2016, evaluation included a complete blood count, nutritional assessment, and testing for parasitic infections, focusing on the primary outcome of anemia, defined as hemoglobin (Hb) < 11 g/dL. The average age at assessment was 20.5 ± 7 months. Ninety-five percent had a lifetime average Hb in the anemic range. Adjusting for age and gender, prior or current malaria infection (prior: Hb β = −0.99, 95% CI: −1.49 to −0.49, *P* = 0.01), or having any current infection with hookworm, *Trichuris*, *Strongyloides*, *Ascaris*, and/or malaria (β = −0.84, 95% CI: −1.36 to −0.33, *P* = 0.01) was associated with decreased current Hb. Nutritional evaluation revealed that children with a declining Hb ate fewer vitamin-A–rich vegetables per week (*P* = 0.01) or eggs (*P* = 0.01), drank more milk (*P* = 0.07), and ate more bread (*P* = 0.01), and were more likely to live in a household that experienced food shortage (*P* = 0.05). The high prevalence of anemia, polyparasitism, and dietary insufficiency among children in rural coastal Kenya suggests that remedial interventions will need to address both diet and parasitic infections to effectively combat this significant health threat.

## INTRODUCTION

Anemia has a strong influence on children’s mental and physical development, including learning, memory, and mental processing speed.^1,2^ This impact has lasting effects through mid-childhood on school performance, whereas adult anemia can lead to detrimental impacts on pregnancy outcomes and work capacity.^3^ Reports in the 1990s estimated that 40% of the world’s population were anemic.^4^ More recent studies have documented partial improvement, with estimates of 25% of the world’s population affected by anemia, accounting for 8.8% of global disability in 2010.^4,5^

Total body iron deficiency is a major contributor to anemia worldwide.^5^ However, not all anemias are due to nutritional iron deficiency. Other causes include dietary insufficiencies of folate and vitamin B_12_, acute blood loss from parasitic infection, chronic hemolysis, thalassemia and other hemoglobinopathies, and anemia of chronic inflammation.^6–9^ The role of parasitism in anemia has been addressed in recent articles,^10–13^ with anemia specifically linked to certain parasite infections, including malaria,^12,14,15^ hookworm,^11,16^ and *Schistosoma* infection.^7^ Anemia of chronic inflammation with hepcidin-associated iron sequestration is another established cause of chronic anemia in parasite-endemic areas,^15,17^ but low dietary iron intake and impaired nutritional status in the same at-risk locations can yield mixed patterns of iron-deficiency + inflammation-related anemia.^18–20^

In 2004, a WHO and CDC joint meeting emphasized the importance of determining the contribution of iron deficiency to anemia before treating with iron supplementation.^6,21^ Epidemiological evidence suggested that iron deficiency and/or sequestration might provide a protective effect against lethal malaria during early infancy.^15,22^ There has been concern that routine iron supplementation could actually prove detrimental in certain settings.^23^ However, recent meta-analysis of randomized clinical trials indicates that iron treatment does not increase the risk of clinical malaria when regular malaria prevention or management services are provided,^24^ and subsequent WHO guidelines recommend iron supplementation in children along with prevention, diagnosis, and treatment of malaria.^25^ As a result, there is an urgent need to detail the prevalence, severity, and mixed etiologies of anemia in at-risk pediatric populations, so that appropriate interventions can be devised for areas where iron supplementation and intermittent preventive therapy for malaria (IPTi) are only inconsistently available. For the present study, we hypothesized that past or present parasitic infections of the child (or the mother while the child was in utero), or current dietary practices of the child are significant contributors to the anemia in children aged 0.5–3 years. A complete blood count (CBC) and nutrition questionnaire, with data on parasitic infection, was used to identify patterns of microcytic and macrocytic anemias and their association to maternal and infant dietary habits and infectious burden.

## METHODS

### Ethical approval and participant eligibility.

This observational cross-sectional study was a sub-study of a longitudinal maternal-child cohort study on the effects of parasitism on childhood vaccine responses (Bill & Melinda Gates Foundation Healthy Growth Award; Enhancing Infant Immunity: Effect of Early Maternal Treatment for Parasitic Infections; PI: Charles King, MD) (Supplemental Material 1).^26^ Ethical approval was provided by the Kenyatta National Hospital/University of Nairobi Ethics and Research Committee, and by the Institutional Review Boards for Human Subjects Research at Stanford University and Case Western Reserve University. Briefly, pregnant women had to be at least 15 years old, to be willing to be tested for parasitic infections during antenatal visits and at delivery, and to deliver and receive prenatal and postnatal care at the Msambweni County Referral Hospital in coastal Kenya. They further had to agree to have their child examined at 10 and 14 weeks, at 6 months of age, and every 6 months thereafter until the child was 3 years old. The mothers provided written informed consent for examination and testing for themselves and their children, and could enroll during the second or third trimester of their child’s pregnancy. Following delivery, infants were excluded from the cohort if they and were born at < 36 weeks gestation (prematurity) or small for gestational age, defined as birth weight less than 1500 g. During their participation, infants experiencing illness were evaluated by study clinical caregivers and treated as needed for any active health problem. Iron supplementation and IPTi were not available and not routinely given before the present cross-sectional study.

### Study design.

Data for the present anemia study were collected from cohort children, aged 0.5–3 years, when they attended their scheduled follow-up visits between June through August 2016. Testing was performed at that time for detection of anemia, concurrent parasitism, and dietary insufficiencies or pica. Results were evaluated according to demographic characteristics of the mother, as well as parasitic infection status, hemoglobin (Hb) levels, and physical characteristics that had been measured during previous cohort protocol visits.

### Laboratory tests.

Before the 2016 survey, Hb values were measured using finger-prick blood in a HemoCue spectrophotometer (HemoCue, Ångelholm, Sweden).^27^ For the 2016 anemia study, an automated CBC was performed using the automated hematology analyzer (Nihon Kohden model: MEK 7222 K, Celltac E, Tokyo, Japan) on 0.5 mL samples of ethylenediaminetetraacetic acid (EDTA)-anticoagulated whole blood taken by venipuncture. Complete blood count testing was performed onsite at the Msambweni County Referral Hospital Clinical Hematology laboratory on the same day blood was collected.

Using the CBC parameters, anemia was defined as Hb < 11 g/dL and severe anemia as Hb < 9 g/dL, according to WHO/CDC definitions for children aged 6 months to 5 years.^6^ Reference values for normal mean corpuscular volume (MCV) and mean corpuscular Hb concentration (MCHC) were as follows: MCV > 70 fL and MCHC > 30.0 g Hb/dL for children aged 6 months to 2 years, and MCV > 75 fL and MCHC > 31.0 g Hb/dL for children aged 2–6 years.^28^ The normal red cell distribution width (RDW) range used for children younger than 2 years is not yet determined, and the WHO range of 11.5–14.5% for children older than 2 years was used as a reference for the younger children.^6^

During pregnancy, at delivery, and at each child follow-up visit, malaria was detected by polymerase chain reaction/ligation detection reaction (PCR/LDR) assay or a malaria blood smear, and infection was defined as a positive result by either test.^29,30^ Intestinal parasites were detected by microscopic stool analysis for ova using the Ritchie method.^31^ *Schistosoma haematobium* infection was detected by both standardized urine filtration for microscopic detection of eggs and ELISA detection of antiparasite IgG4 antibodies in plasma; subjects were defined as infected if either test was positive.^10,32,33^ Mothers and children found to have malaria and/or intestinal helminths were appropriately treated within 72 hours, mothers were also treated with IPTp and had access to bed nets.

Children’s exposure to parasitic infection was scored during their gestational period based on documented maternal antenatal infections; postnatal infections were determined based on all cohort visits before the present study’s 2016 visit. Current exposure was determined by testing at the 2016 study visit. “Full infancy” was defined as combined postnatal and current status, taken together. Soil-transmitted helminthiasis (STH) was defined as having hookworm, *Ascaris*, *Trichuris*, and/or *Strongyloides* infection. “Any Infection” referred to having STH, malaria, and/or *S. haematobium.* The Msambweni location is endemic for urogenital schistosomiasis and the local rates of *S. haematobium* infection prevalence among school-age children (5–18 years old) are more than 60%.^34^ Children with missing infection data, largely because of absent stool samples, were removed pairwise from the analyzed groups (Supplemental Material 2).

### Study procedures.

Anthropometric measurements were taken on children at each visit by specially trained staff and *Z*-scores were computed using the WHO Anthro software (WHO, Department of Nutrition for Health and Development, Geneva, Switzerland). Age-adjusted body mass index (BMI), height-for-age, and weight-for-age *Z*-scores were interpreted as wasting, stunting, and underweight, respectively, when these *Z*-scores were < −2.

A nutritional assessment based on dietary intake was conducted with each mother regarding her child at the 2016 study visit. The questionnaire gathered information about the frequency that the child consumed certain common foods. It also assessed family food security (through a modified Household Food Insecurity Access Scale [HFIAS] questionnaire), the prevalence of locally common slate-chewing practices during pregnancy, of children’s pica, and whether any immediate family members had sickle-cell disease (Supplemental Material 3).^35^

### Statistical methods.

Statistical analyses were performed using SAS version 9.4 (SAS Institute Inc., Cary, NC) and R Studio version 1.0.136 (R Core Team [2014]. R: A language and environment for statistical computing. R Foundation for Statistical Computing, Vienna, Austria. URL http://www.R-project.org/). Statistical significance of group differences was determined using *t*-test, Fisher’s exact test, or Chi-square testing, as appropriate. Outlier Hb values (Hb > 16) only occurred with HemoCue and were removed from analysis and treated as a missed follow-up data. Multiple imputation (PROC MI in SAS 9.4) was used to impute missing infection status data for all parasites. The fully conditional specification method allowed for imputation on a variable-by-variable basis.^36^ Each missing value was imputed five separate times with age, gender, average Hb, and presence of other infections at each time period as conditional predictors. All five imputed datasets were pooled and used in the linear mixed model of current Hb and regressed individually on each parasitic infection at each time period while adjusting for age and gender. Each β estimate was calculated with current Hb regressed on each food type and parasitic infection while adjusting for age and gender, with significant values *P* < 0.05 and adjustment for false discoveries using the Benjamini–Hochberg method.^37^ Percent variation and variable importance were assessed using the randomForest package to present %IncMSE (percent increase in mean square error) calculated as the mean square difference between models if that variable was removed and replaced with a random variable.^38^

## RESULTS

### Demographics.

There were 244 participants during the 2016 survey, of whom 102 (42%) were male and 185 (76%) were found to be anemic at the time of their visit (Table 1). Consistent with findings for a previous 2006–2009 infant cohort from the same location,^39^ the mean age for anemic children was significantly younger than that of non-anemic children (*P* < 0.001) (Table 1). There were two children with sickle-cell disease, one of whom was anemic. There were 18 (7%) children who had a first-degree family relation (parent or sibling) diagnosed with sickle-cell disease; of these study children, 13/18 (72%) had anemia. The average recorded maternal Hb at the time of a child’s delivery was significantly lower among children who were anemic in 2016, irrespective of the child age (*P* < 0.01) (Table 1).

**Table 1:** Demographic data for the 244 children enrolled in the anemia study

Anemia status in summer 2016				
Child characteristics	Total cohort *n* = 244 (100)	Non-anemic *n* = 59 (24)	Anemic *n* = 185 (76)	*P*-value
Age in summer 2016, months (±SD)	20.55 ± 6.92	24.27 ± 6.47	19.35 ± 6.64	< 0.001*
Gender, *n* (%)				
Female	142 (58)	37 (63)	105 (57)	0.40†
Maternal age, years (±SD)	26.63 ± 6.15	27.03 ± 6.37	26.49 ± 6.09	0.56*
Maternal Hb at delivery, g/dL (±SD)	9.78 ± 1.79	10.37 ± 1.56	9.60 ± 1.83	0.007*
Cord blood Hb, g/dL (±SD)	13.95 ± 3.08	13.73 ± 2.88	13.87 ± 2.76	0.76*
Maternal occupation *n* (%)				0.14‡
Housewife	171 (70.1)	41 (69.5)	130 (70.3)	1.00*
Others	31 (12.7)	10 (16.9)	21 (11.4)	0.37*
None	29 (11.8)	8 (13.6)	21 (11.4)	0.82*
Unknown	13 (5.3)	0 (0.0)	13 (7.0)	0.08‡
Number of previous pregnancies	2.72 ± 1.65	2.51 ± 1.49	2.78 ± 1.71	0.32*
Maternal HIV+ *n* (%)	14 (5.7)	5 (8.5)	9 (4.9)	0.47‡
Sickle cell history, *n* (%)				
Child	2 (0.8)	1 (1.7)	1 (0.5)	0.43‡
Family	18 (7.3)	5 (8.5)	13 (7.0)	0.93‡
Complete blood count, *n* (%)				
Microcytic	179 (73.7)	29 (49.2)	150 (81.5)	< 0.001†
Hypochromic	24 (9.9)	0 (0)	24 (13.0)	0.008‡
Anisocytosis	131 (53.7)	13 (22.0)	118 (63.8)	< 0.001†
Current infection, *n* (%)				
Malaria	12 (4.9)	1 (1.7)	11 (5.9)	0.30‡
STH	23 (12.1)	3 (6.1)	20 (14.2)	0.20‡
Malaria or STH	34 (13.9)	4 (6.8)	30 (16.2)	0.08‡
Malaria and STH	1 (0.4)	0 (0)	1 (0.5)	1.00‡
Any infection, *n* (%)				
Antenatal	105 (43.0)	27 (51.9)	78 (48.1)	0.75†
Postnatal	67 (27.5)	13 (22.0)	54 (29.2)	0.37†
Current	34 (13.9)	4 (6.8)	30 (16.2)	0.11†
Full infancy	67 (27.5)	13 (22.0)	54 (29.2)	0.37†
Nutritional proxies, *n* (%)				
Wasting	12 (4.9)	2 (3.4)	10 (5.4)	0.74‡
Stunting	60 (24.6)	11 (18.6)	49 (26.5)	0.30†
Underweight	19 (7.8)	6 (10.2)	13 (7.0)	0.61†

Hb = hemoglobin; STH = soil-transmitted helminthiasis. The children were stratified by their anemia status during their 2016 study visit with a threshold for “anemic” of < 11 g/dL. Hookworm, *Ascaris*, *Trichuris*, and *Strongyloides* infection were included in “any STH” category. See Supplemental Material 2 for evaluation of missing infection data points.

* *t*-test.

† Chi-squared test.

‡ Fisher’s exact test.

### Anemia prevalence and trends.

The CBC testing characterized the MCV, MCHC, and RDW in each sampled child’s blood during the 2016 study period. There was significantly more microcytosis, defined as low MCV (82% for anemic children versus 50% for non-anemic children); hypochromatic red blood cells, defined as low MCHC (24% versus 0%); and diverse red blood cell sizes, defined as high RDW (64% versus 22%), in the currently anemic compared with the non-anemic group (Table 1).

**Figure 1.**
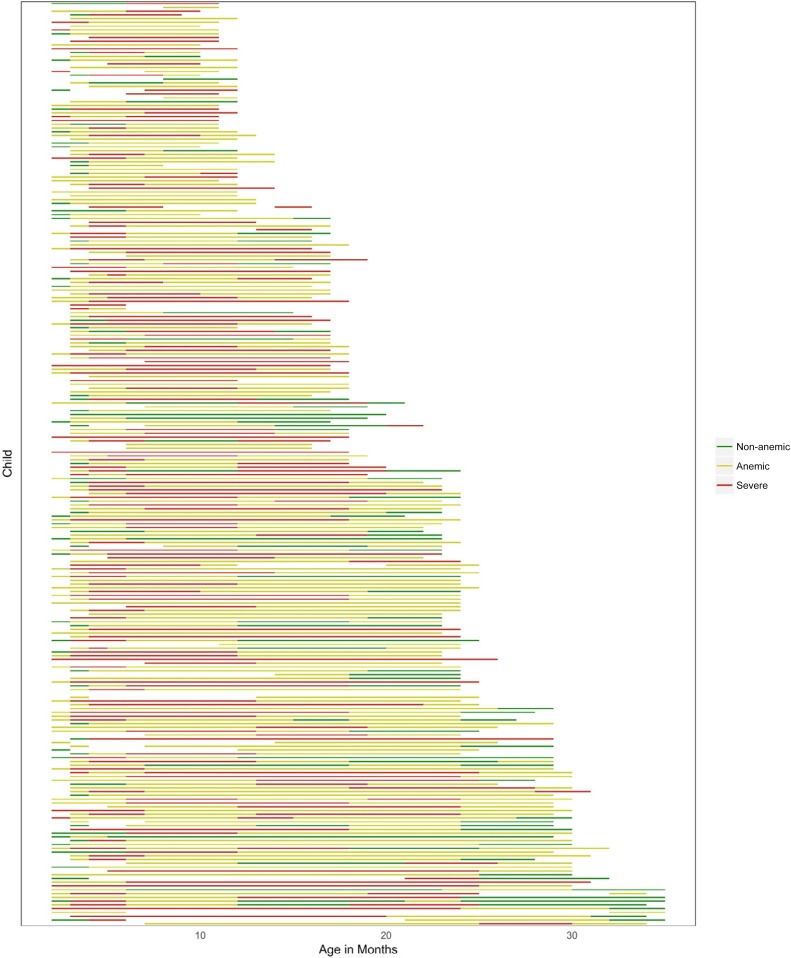
The line plot shows the individual anemia status over the cohort follow-up period for each child from visit 1 (2.5 months old) to their 2016 visit during the present anemia study (1–3 years old). “Non-anemic” (green) is hemoglobin (Hb) ≥ 11 g/dL, “anemic” (yellow) is 9 g/dL ≤ Hb < 11 g/dL, and “severe” (red) is Hb < 9 g/dL.

Longitudinal Hb measurements on individual children are graphically portrayed in Figure 1, which shows that only one child remained anemia free during infancy and early childhood. Just 11 (5%) children had an average Hb across all their visits that was non-anemic.

### Anemia and anthropometrics.

Anthropometric *Z*-scores were assessed and compared with concurrent Hb results during the 2016 study visit (Table 1). Stunted (5% anemic versus 3% non-anemic), wasted (27% versus 19%), and underweight (7% versus 10%) children were common in both the anemic and non-anemic groups, with no statistically significant differences. When stunting was further investigated, there was no significant difference in anemia between the stunted versus non-stunted children; currently, stunted children were anemic at 79.5% of their lifetime visits compared with 75.5% of visits in the not currently stunted group.

### Anemia and infection status.

**Figure 2.**
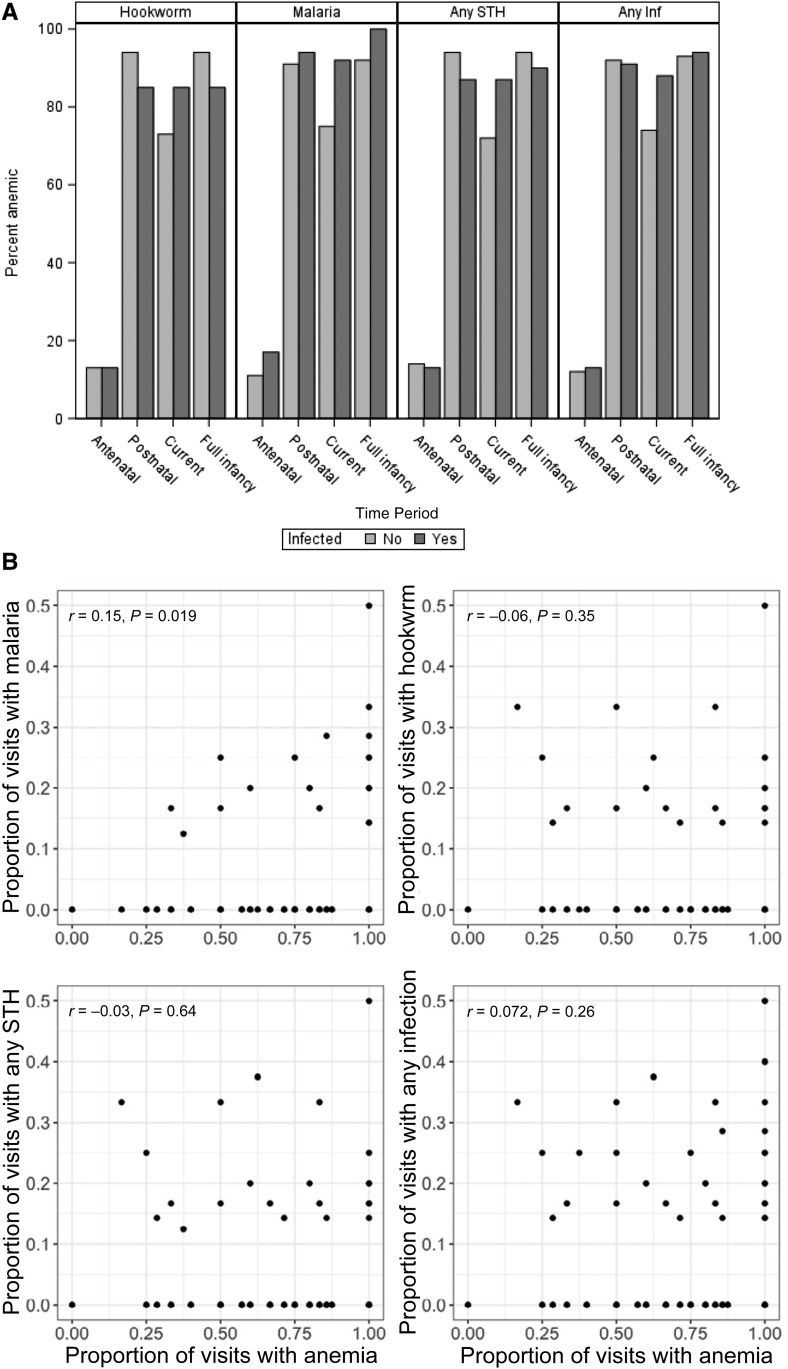
(**A**) Anemia status by exposure to parasitic infection during time periods of “antenatal” (defined as maternal infection during pregnancy), “postnatal” (defined as postnatal period before current 2016 visit), “current” (2016 visit), or “full infancy” (defined as postnatal + current). (**B**) Children’s percent visits with anemia graphed against their percent visits with any specific infection or group of infections. Correlation coefficient is calculated as Spearman’s rho. Hookworm, *Ascaris*, *Trichuris*, and *Strongyloides* were included in the “any soil-transmitted helminthiasis (STH)” category. “Any infection” included STH, malaria, and *Schistosoma haematobium*. Missing data information can be found in Supplemental Material 2.

Parasitic infections were highly prevalent in this child cohort, with both anemic and non-anemic children infected. All children who were currently infected within a category (malaria, hookworm, any STH, or any infection) had prior childhood infections within that category. With regard to antenatal maternal infection, antenatal infection was identified in 30% of those with current hookworm, 33% of those with current malaria, 40% of those with any STH, and 47% of those with any infection. Anemia status was linked to specific infection categories at each certain time interval (Figure 2A). Malaria was consistently associated with anemia, and, although not significant, the children with antenatal maternal malarial infection were currently anemic at higher rates than those without antenatal maternal malarial infection. Figure 2B portrays the correlation between a child’s frequency of anemic visits and his or her frequency of visits with infection(s). This association was strongest and significant for the frequency of malaria episodes (*r* = 0.15, *P* = 0.019).

**Figure 3.**
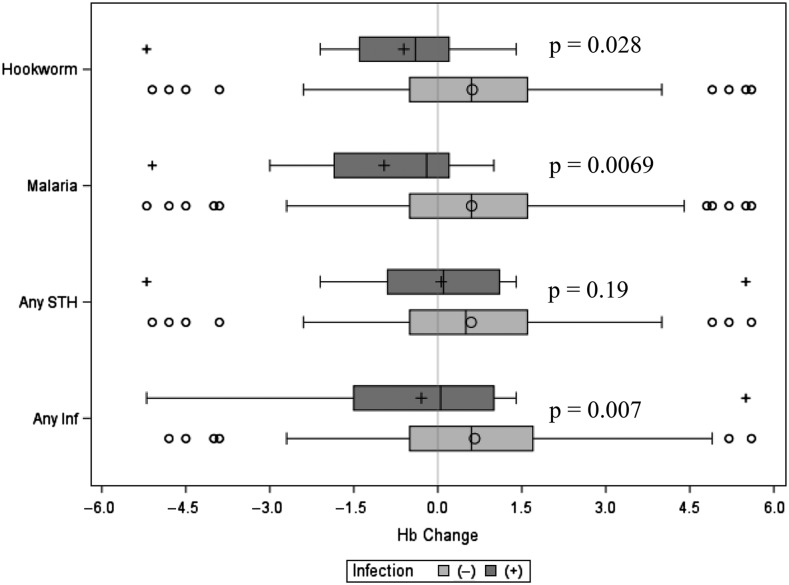
Box plots of infection status with hemoglobin (Hb) change, defined as the difference between current Hb and Hb immediately before the 2016 visit, contrasted by infection status. Black lines represent the median change, boxes reflect the 25% to 75% interquartile range, whiskers represent the 5th–95th percentile range, and the circles and pluses represent infection (+) and no infection (◯), respectively. *P*-values represent Hb differences between infection and no infection groups within a designated category.

To further assess the impact of current parasitic infection on Hb, the difference between the current Hb and the Hb during the visit immediately prior was calculated and compared with the child’s parasitic exposure status. The children were stratified based on infection status for each parasite or group of parasites and the average change in Hb was computed for each status group (Figure 3). It is apparent that, compared with non-infected children, children currently infected with hookworm, malaria, or any infection (malaria, hookworm, *Trichuris*, *Ascaris*, *Strongyloides*, and *S. haematobium*) had a statistically significant recent relative decline in Hb (Figure 3).

### Anemia and nutrition.

**Figure 4.**
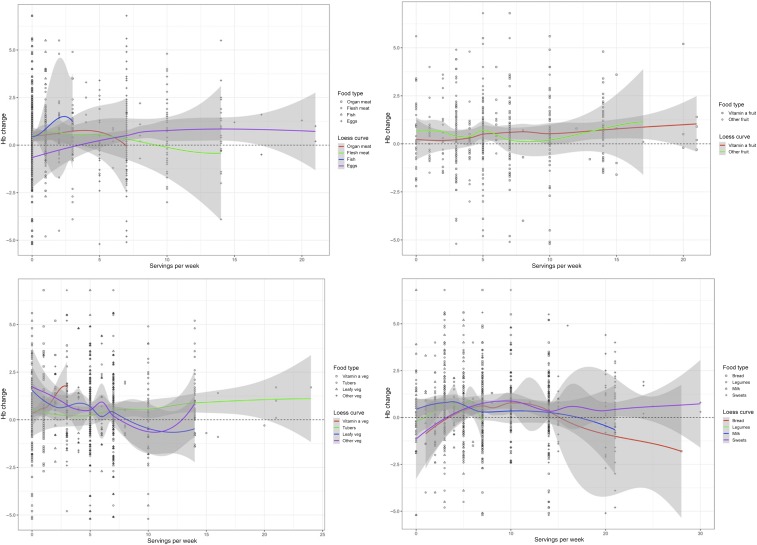
Scatter plots and Loess curves categorized by food types comparing number of servings per week reported by nutrition questionnaire and hemoglobin (Hb) change from prior visit.

The nutritional assessment queried food security, breastfeeding, antenatal maternal slate chewing, child pica, and frequency of eating specific foods (Table 2, Supplemental Materials 3 and 4). Typically, the children were exclusively breastfed for 3 months, then porridge was introduced, and by 6 months, they had completely transitioned to solid foods. Of the children in the study, 21/244 (9%) were breastfed for less than 6 months, and 16 of these 21 children (76%) who were breastfed for less than 6 months were anemic at the “current” visit. None of the children in the study were breastfeeding at the time of the nutritional assessment. All children whose mothers answered yes to 11 or 12 of the 12 total food security questions were anemic at the “current” time point (Supplemental Material 4A). Seventy percent of mothers reported making smaller portions for their children’s meals and 62% said their child was not eating enough food. Of mothers who responded yes to selected food security questions, about 75% of those corresponding children were anemic (Supplemental Material 4B). Children who had a decline in Hb when compared with visits before 2016 ate less vitamin-A-rich vegetables and eggs, more milk and bread, and lived in a household that experienced significant food shortage in the previous year (Table 2, Figure 4). Maternal slate chewing during pregnancy and child pica were also more frequent in the group exhibiting a recent decline in Hb (Table 2).

**Table 2:** Nutrition and dietary reports according to current anemia or changing Hb status: 244 children were stratified by their anemia status during their 2016 visit with a cutoff threshold for “anemic” of < 11 g/dL or were stratified based on the most recent change in their Hb levels (decline, no change, or improvement)

Nutrition table	Anemia status in summer 2016				Anemia status change			
	Total cohort (*n* = 244 [100])	Non-anemic (*n* = 59 [24])	Anemic (*n* = 185 [76])	*P*-value	No change (*n* = 23)	Decline (*n* = 80)	Improve (*n* = 140)	*P*-value
Food security, *n* (%)								
Little variety	160 (66.1)	37 (64.9)	123 (66.5)	0.83*	15 (71)	52 (68)	84 (65)	0.79*
Household ran out	48 (19.8)	11 (19.3)	37 (20.0)	0.91*	4 (19)	22 (29)	19 (15)	0.05*
Child not healthy	95 (39.3)	23 (39.7)	72 (39.1)	0.94*	11 (52)	27 (36)	53 (40)	0.37*
Child not enough food	151 (62.4)	37 (64.9)	114 (61.6)	0.65*	15 (71)	50 (65)	77 (59)	0.47*
Smaller portions	171 (70.7)	41 (71.9)	130 (70.3)	0.81*	14 (67)	57 (74)	89 (68)	0.65*
Dietary practices								
Breastfed, *n* (%)	244 (100)	59 (100)	185 (100)	–	21 (100)	77 (100)	132 (100)	
Duration of breastfed in months, mean (±SD)	5.88 ± 1.11	5.85 ± 0.74	5.89 ± 1.21	0.77†	6.24 ± 2.84	5.87 ± 0.73	5.89 ± 0.61	0.33†
All foods introduced by age 1, *n* (%)	206 (84.7)	51 (87.9)	154 (83.2)	0.12‡	13 (62)	117 (89)	62 (81)	0.02*
Age at which first foods introduced (in months), mean (±SD)	2.91 ± 0.36	2.93 ± 0.25	2.90 ± 0.38	0.42†	2.86 ± 0.57	2.91 ± 0.33	2.93 ± 0.25	0.59†
Slate chewing during pregnancy, *n* (%)	131 (53.5)	27 (45.8)	104 (56.2)	0.16*	11 (52)	48 (62)	60 (45)	0.06*
Child eats nonedible food, *n* (%)	97 (39.8)	19 (32.2)	78 (42.2)	0.17*	9 (43)	37 (48)	41 (31)	0.04*
Food type eaten per week (mean ± SD)								
Bread	9.54 ± 3.51	9.51 ± 3.05	9.55 ± 3.65	0.94†	8.38 ± 3.71	10.46 ± 4.00	9.30 ± 3.14	0.01†
Vitamin-A vegetables	0.43 ± 0.73	0.54 ± 0.79	0.39 ± 0.70	0.17†	0.52 ± 0.93	0.23 ± 0.51	0.57 ± 0.79	0.01†
Tubers	8.35 ± 4.03	8.32 ± 4.01	8.36 ± 4.05	0.95†	8.24 ± 4.31	7.92 ± 4.08	8.64 ± 4.06	0.47†
Leafy vegetables	5.57 ± 2.15	5.17 ± 1.86	5.70 ± 2.23	0.10†	4.81 ± 1.36	6.08 ± 2.43	5.37 ± 2.06	0.02†
Other vegetables	5.24 ± 1.86	4.97 ± 1.93	5.33 ± 1.83	0.19†	4.90 ± 1.30	5.48 ± 2.11	5.10 ± 1.80	0.27†
Vitamin-A fruits	8.03 ± 4.53	8.19 ± 4.38	7.98 ± 4.59	0.77†	7.76 ± 4.66	7.44 ± 4.21	8.65 ± 4.74	0.17†
Other fruits	4.02 ± 2.83	4.00 ± 3.41	4.03 ± 2.63	0.94†	4.19 ± 3.01	3.95 ± 2.48	4.01 ± 2.99	0.94†
Organ meat	0.24 ± 0.86	0.36 ± 1.08	0.21 ± 0.77	0.24†	0.14 ± 0.48	0.18 ± 0.88	0.27 ± 0.87	0.67†
Flesh meat	1.24 ± 1.66	1.53 ± 1.71	1.15 ± 1.64	0.13†	0.76 ± 1.14	1.03 ± 1.41	1.48 ± 1.86	0.06†
Eggs	0.20 ± 0.47	0.27 ± 0.55	0.17 ± 0.43	0.16†	0.14 ± 0.36	0.08 ± 0.27	0.27 ± 0.55	0.01†
Fish	8.02 ± 3.10	8.10 ± 2.58	7.99 ± 3.25	0.82†	7.24 ± 2.45	7.96 ± 3.62	8.27 ± 2.98	0.36†
Legumes	3.67 ± 1.59	3.64 ± 1.41	3.68 ± 1.65	0.88†	3.33 ± 2.06	3.68 ± 1.81	3.67 ± 1.42	0.66†
Milk	5.41 ± 4.44	4.78 ± 3.34	5.61 ± 4.73	0.21†	5.95 ± 5.20	6.09 ± 5.14	4.70 ± 3.72	0.07†
Sweets	14.64 ± 5.70	14.86 ± 5.64	14.57 ± 5.74	0.73†	14.05 ± 6.02	15.14 ± 5.87	14.31 ± 5.51	0.54†

Hb = hemoglobin. *P*-values represent differences between the groups.

* Chi-squared test.

† *t*-test.

‡ Fisher’s exact test.

### Adjusting for age and gender.

Analysis of current Hb levels was assessed combining parasitic infection status and nutritional intake assessment, while adjusting for age and gender in linear regression models (Table 3). The analysis suggested that malaria had the most significant independent association with decline in Hb. Antenatal maternal malaria infection was associated with a decrease in Hb, β = −0.52 g/dL ([−1.04 to −0.01]; *P* < 0.04) that lost significance when an false discovery rate (FDR)-adjusted *P*-value was calculated using the Benjamini–Hochberg method (FDR-adjusted *P* = 0.29). Postnatal malaria infection was associated with a decreased Hb, β = −0.99 g/dL ([−1.49 to −0.49]; FDR-adjusted *P* = 0.01), and current malaria infection with a decreased Hb, β = −1.76 g/dL ([−2.58 to −0.94]; FDR-adjusted *P* < 0.001) (Table 3). Antenatal malaria infection and childhood anemia were further explored with an additional multivariate model that adjusted for age, gender, and maternal malaria infection at 6-month intervals. Although antenatal malaria infection was significantly associated with a Hb drop at the “current” time point, it was not associated with a significant decrease when values at a specific age were tested for significance (Supplemental Material 5).

**Table 3:** Analysis of infection and nutritional practices

Model estimates			
Variables	Adjusted for age and gender		
	β estimate (95% CI)	*P*-value	FDR adjusted *P*-value
Model for current Hb (Hb during summer 2016 visit)			
Malaria			
Antenatal	−0.52 (−1.04, −0.01)	0.04	0.29
Postnatal	−0.99 (−1.49, −0.49)	0.001	0.01
Current	−1.76 (−2.58, −0.94)	< 0.001	< 0.001
Hookworm			
Antenatal	0.17 (−0.26, 0.61)	0.42	0.84
Postnatal	0.15 (−0.43, 0.73)	0.6	0.89
Current	−0.47 (−1.50, 0.57)	0.36	0.83
*Trichuris*			
Antenatal	−0.27 (−0.92, 0.38)	0.4	0.84
Postnatal	0.21 (−0.74, 1.16)	0.67	0.89
Current	−0.91 (−2.43, 0.61)	0.22	0.73
*Ascaris*			
Antenatal	−0.20 (−1.56, 1.15)	0.77	0.95
Postnatal	−0.07 (−1.11, 0.97)	0.9	0.97
Current	−0.28 (−1.64, 1.09)	0.69	0.89
*Strongyloides*			
Antenatal	0.06 (−0.85, 0.97)	0.89	0.97
Postnatal	0.06 (−1.78, 1.89)	0.95	0.97
Current	−0.66 (−3.93, 2.62)	0.69	0.89
Any soil-transmitted helminthiasis			
Antenatal	0.11 (−0.28, 0.51)	0.58	0.89
Postnatal	−0.01 (−0.52, 0.51)	0.97	0.97
Current	−0.61 (−1.24, 0.02)	0.06	0.29
Any infection			
Antenatal	−0.05 (−0.44, 0.33)	0.78	0.95
Postnatal	−0.54 (−0.94, −0.14)	0.01	0.11
Current	−0.84 (−1.36, −0.33)	0.001	0.01
Nutrition model for current Hb (continuous variable food type per week)			
Bread	−0.01 (−0.06, 0.05)	0.8	0.95
Vitamin-A vegetables	0.18 (−0.08, 0.43)	0.18	0.66
Tubers	0.02 (−0.02, 0.07)	0.31	0.80
Leafy vegetables	−0.05 (−0.14, 0.03)	0.24	0.73
Other vegetables	−0.09 (−0.19, 0.00)	0.06	0.29
Vitamin-A fruits	0.01 (−0.03, 0.05)	0.55	0.89
Other fruits	−0.04 (−0.10, 0.03)	0.25	0.73
Organ meat	−0.01 (−0.23, 0.21)	0.94	0.97
Flesh meat	−0.03 (−0.15, 0.09)	0.65	0.89
Eggs	0.19 (−0.21, 0.59)	0.35	0.83
Fish	−0.04 (−0.10, 0.02)	0.15	0.66
Legumes	−0.04 (−0.16, 0.07)	0.46	0.88
Milk	−0.04 (−0.08, 0.00)	0.05	0.29
Sweets	−0.01 (−0.04, 0.02)	0.66	0.89

Hb = hemoglobin. β estimates of infection status along with 95% CIs, P-values, and FDR-adjusted P-values are listed. Each β estimate was calculated with current Hb regressed on each food type while adjusting for age and gender.

### Variable importance.

**Figure 5.**
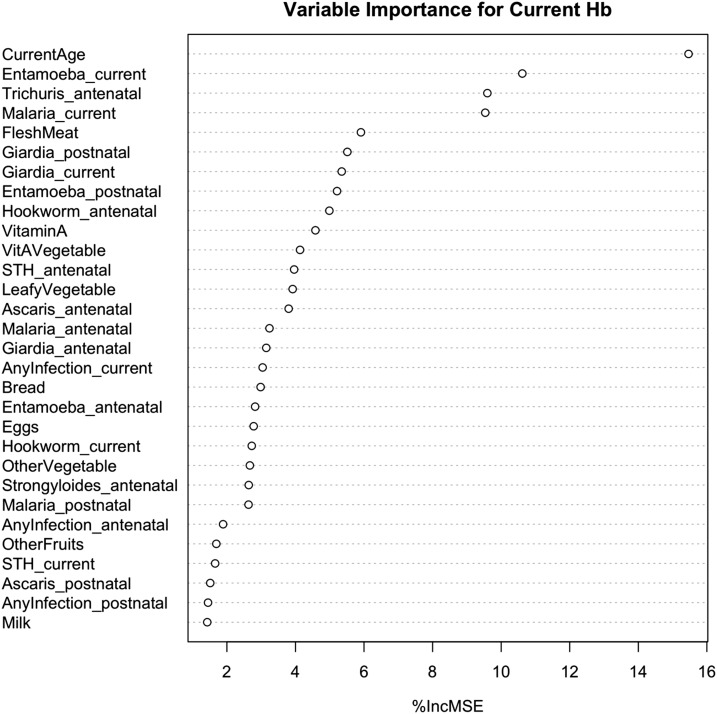
Variable importance ranked based on impact on current hemoglobin (Hb) level. Percent increase in mean square error (%IncMSE) calculated as the mean square difference between models if that variable was removed and replaced with a random variable. The listed variables accounted for 18.07% of variation in current Hb.

To further assess the impact of each variable on the current Hb level, a %IncMSE was calculated and was found to account for 18.07% of variation within the data (Figure 5). It is apparent that current age has the strongest impact on current Hb with individual STH and malaria close behind.

## DISCUSSION

Hb values from a CBC during our 2016 study of infants from rural coastal Kenya found that 76% of children were anemic with predominantly microcytic, hypochromic red cells with anisocytosis. Of the 244 children, only one had non-anemic Hb values for his/her entire life; all other children were anemic during at least one follow-up visit and 95% of the children had an average Hb over all study visits that was anemic. For many children, the Hb values decreased after birth, likely secondary to physiologic anemia of the newborn, and only some children recovered from their anemic state during their period of follow-up. Similar early life Hb trajectories were observed in an earlier infant cohort study in the same location in 2006–2009^39^ and variable importance modeling validates these findings by suggesting current age has the strongest impact on current Hb. Statistical modeling indicated the significant contribution of malaria infection on the Hb level and the current anemia status of these children. When further investigated, antenatal malarial infection was not significantly associated with a drop of Hb across 6-month intervals in infancy, which implied that perhaps the correlation with “current” anemia and antenatal infection might be due to joint high-risk mother and child exposure to malaria rather than a specific maternal-fetal interaction in utero. This correlation suggests that treating and preventing malaria by reducing exposure risk in the mother’s environment may further protect the child from current anemia.

Our findings further validate the correlation between malarial infection and childhood anemia in southeastern Kenya.^8^ Current STH infection was also associated with significantly diminished current Hb, and any infection in the postnatal or current period was linked to lower Hb levels. Together, these data emphasize the need to treat and prevent parasitic infections, during both pregnancy and early childhood, to limit the anemia of early childhood.

Nutritional intake and dietary practices also contribute to anemia.^18,19^ Our results show that consumption or nonconsumption of specific foods was correlated with a decline in Hb and a child’s anemia (e.g., consumption of fewer vitamin-A–rich vegetables and eggs and of more milk and bread). This dietary link may be explained by the established roles of calcium and white flour in interfering with iron absorption.^40^ In addition, food insecurity was likely a contributing factor to childhood anemia.^18^ In our study population, food shortage was common and not limited to the anemic or declining Hb groups of children. This lack of food quantity is also likely to be a substantial factor contributing to the local high prevalence of growth stunting.

Geophagy and pica were common among pregnant women and children in this cohort, but it is unclear if these practices were a cause or a result of anemia. Previous studies have described the potential impact these practices on the bioavailability of iron, which would further increase the risk of anemia.^41,42^ Declining Hb was significantly associated with increased maternal slate chewing and pica, suggesting that the nutritional deficits span not only quality and quantity of food availability but also include the late impact of maternal geophagy.

A previous study conducted in nearby Jego, Kenya, among children aged 3–11 years with high rates of parasitic infections found that childhood anemia prevalence was 79% and asymptomatic malaria infection was one of the strongest correlates.^8^ That study concluded that anemia of inflammation and iron deficiency are both significant sources of childhood anemia, and emphasized the need to address both to properly manage childhood anemia.^8^ However, children younger than 2 years, who are most vulnerable to the deficiency linked to anemia, were not included in that study. Another previous study noted the role of parasitic infection, dietary intake, and genetic diseases on anemia in children ages 6–23 months in south-central Côte d’Ivoire.^19^ That study found that malaria and *Schistosoma* infection, inflammation, and stunting were negatively associated with Hb concentrations and concluded that effective anemia prevention needs to be multifactorial.^19^ Both their study and ours emphasize the need to address a comprehensive group of factors, including dietary deficiencies and parasitic infections, when addressing early childhood anemias.

Combating anemia in this cohort of children must be multidimensional and include iron supplementation as well as parasitic infection treatment and prevention in the child and mother. Scott et al.^43^ used a logistic regression meta-analysis of anemia studies in the pediatric population younger than 5 years in Africa and found that a 1 g/dL Hb increase would decrease the risk of child mortality by 24%, suggesting that approximately 1.8 million deaths could be avoided by increasing Hb values. However, coordinated interventions must be used; delivery of iron alone may prove detrimental in the face of inadequate malaria prevention, and iron ingestion may prove ineffective in the face of chronic inflammation.^15,22,23^ Therefore, preventive treatment for both anemia and malaria must occur simultaneously. Safe improvement in Hb levels has the potential to appreciably decrease child mortality and increase the quality of life for the children who are treated.

There are limitations to the present study. These include its relatively short duration, which allowed only 244 of the 598 children in the parent study’s cohort to be included. There were also missing infection data on STH because of absent stool samples. However, we believe that the multiple imputation method used in our analyses provided the best estimation of likely infection status where there were missing values (Supplemental Material 2). Other limitations to the study included lack of information on reticulocytosis, serum ferritin, and soluble transferrin receptor, which were not available in our laboratory. However, it is known that children aged 3–5 years in a nearby town had significantly lower MCV, iron, and serum iron-binding saturation levels than older children, suggesting that early life iron deficiency is common in this region.^8^ Hemoglobinopathies and thalassemias that can also cause anemia, particularly in malaria-endemic areas, were outside the scope of this project.^4,44^ It was also outside the scope of the project to test the child’s HIV status, another potential confounder, and ITPi was not used, which may have been a modifying factor. Fourteen of the 244 children (6%) were born to mothers known to be HIV positive at the time of their study enrollment during pregnancy.^44,45^

The results of this study emphasize the pressing need to address anemia in children in coastal Kenya. Anemia was prevalent in up to 76% of children at the time of their study visit, and in up to 95% of children when all their recorded postnatal Hb values were considered. The anemia in this cohort was associated with childhood exposure to parasitic infection and to antenatal maternal malaria infection. We conclude that both will need to be addressed in attempting to improve early childhood Hb. In addition, this study identified specific dietary correlates of childhood anemia, including inclusion of specific foods, food insecurity, maternal slate chewing, and child pica. Overall, where anemia remains a top public health concern should be addressed from multiple directions to effectively prevent the developmental deficits that may ensue.

## Supplementary Files

Supplemental materials
