# Supplementary material for: Early Childhood Anemia in a Birth Cohort in Coastal Kenya: Links to Infection and Nutrition
Source: Am J Trop Med Hyg. 2019 May 6;101(1):242–52. doi: 10.4269/ajtmh.17-0688 (PMC6609201; doi:10.4269/ajtmh.17-0688)
Supplement: Supplementary file 1 [file tpmd170688.SD1.pdf]

Supplement 1.

Pregnant mothers were included if they were residents of Msambweni and received prenatal and postnatal care at the District Hospital's antenatal clinic. Mother-infant pairs were excluded if they:

a) Experienced a complicated infant delivery which resulted in significant infant morbidity
b) The infant was born less than 36 weeks gestation
c) The mother had known chronic illness
d) The mother had severe anemia (Hb < 6 g/dl) requiring hospitalization
e) The mother had a permanent disability that impeded study participation and/or comprehension
f) The family had plans to relocate after delivery

Mothers completed a detailed questionnaire at enrollment that queried their education level, spouse's occupation, and household income.

Infants were followed from birth every six months until three years of age. Twins were included and analyzed as individuals (< 1% of the cohort). Children were also evaluated during any episodes of acute illness that occurred between scheduled visits. A select group of specially-trained clinical staff performed physical examinations on all infants at delivery and at each scheduled follow-up visit. Following Kenya Ministry of Health guidelines, infants received amodiaquine (if < 5kg) or artemether/ lumefantrine (CoArtem) (if > 5kg) for malarial treatment. Infants were treated with mebendazole for soil transmitted helminth infections.

Supplement 2.

	Anemia Status Prenatal (34 missing Hb cord value)			Anemia Status Postnatal				Current Anemia Status				Anemia Status Full Infancy (mean Hb for entire postnatal, including current)				Maternal HIV Status			
Characteristics (n=244)	Non-Anemic (n=184 (75))	Anemic (n=26 (11))	Total # of Missing Parasite Data	Non-Anemic (n=20 (8))	Anemic (n=224 (92))	p-value	Total # of Missing Parasite Data	Non-Anemic (n=59 (24))	Anemic (n=185 (76))	p-value	Total # of Missing Parasite Data	Non-Anemic (n=17 (7))	Anemic (n=227 (93))	p-value	Total # of Missing Parasite Data	HIV- (n=230 (94))	HIV+ (n=14 (6))	p-value	Total # of Missing Parasite Data
Age - months (mean ± SD)	--	--		6.73 ± 3.03	8.19 ± 2.95			24.34 ± 6.50	19.35 ± 6.64			10.05 ± 3.41	10.44 ± 3.25			20.62 ± 6.82	19.43 ± 8.54		
Gender - n (%) (n= 242)	109 (59)	17 (65)		14 (70)	128 (57)			37 (63)	105 (57)			11 (64)	131 (58)			95 (42)	5 (36)		
Hb measurement - (mean ± SD)	14.62 ± 1.79	8.25 ± 2.12		11.45 ± 0.48	9.56 ± 0.79			11.94 ± 1.01	9.43 ± 1.11			11.42 ± 0.32	9.63 ± 0.78			10.00 ± 1.52	10.66 ± 1.60		
Parasites - n (%) // missing #																			
Malaria	29 (16)	6 (23)	30	2 (10)	33 (15)	0.75b		1 (2)	11 (6)	.30b		0 (0)	35 (15)	.14b		12 (5)	0 (0)	.81b	
Hookworm	52 (36) // 40	8 (36) // 4	76	4 (24) // 3	23 (11) // 8		11	2 (4) // 10	11 (8) // 44		54	4 (25) // 1	23 (11) // 10		11	13 (7) // 52	0 (0) // 2		54
Ascaris	4 (3) // 54	0 (0) // 7	93	2 (12) // 3	7 (3) // 8		11	0 (0) // 10	5 (4) // 44		54	1 (6) // 1	8 (4) // 10		11	5 (3) // 52	0 (0) // 2		54
Trichuris	21 (15) // 49	3 (15) // 6	87	0 (0) // 3	11 (5) // 8		11	1 (2) // 10	6 (4) // 44		54	0 (0) // 1	11 (5) // 10		11	7 (4) // 52	0 (0) // 2		54
Strongyloides	7 (5) // 54	1 (5) // 7	92	0 (0) // 3	3 (1) // 8		11	0 (0) // 10	1 (1) // 44		54	0 (0) // 1	3 (1) // 10		11	1 (1) // 52	0 (0) // 2		54

Giardia	2 (2) // 53	0 (0) // 7	92	3 (18) // 3	23 (11) // 8		11	5 (10) // 10	10 (7) // 44		54	2 (13) // 1	24 (11) // 10		11	12 (7) // 52	3 (25) // 2		54
Entamoeba	51 (36) // 44	6 (30) // 6	82	1 (6) // 3	8 (4) // 8		11	3 (6) // 10	1 (1) // 44		54	2 (13) // 1	7 (3) // 10		11	4 (2) // 52	0 (0) // 2		54
S. *haematobium*	16 (11) // 44	3 (13) // 3	79	0 (0) // 5	0 (0) // 25		30	0 (0) // 59	1 (1) // 184		243	0 (0) // 3	0 (0) // 27		30	0 (0) // 229	0 (0) // 14		243
Any STH	67 (45) // 35	10 (43) // 3	69	5 (29) // 3	34 (16) // 8		11	3 (6) // 10	20 (14) // 44		54	4 (25) // 1	35 (16) // 10		11	23 (13) // 52	0 (0) // 2		54
Any Infection	117 (64)	16 (62)	30	8 (40)	85 (38)	0.86c		12 (20)	40 (22)	0.83c		6 (35)	87 (38)	0.80c		49 (21)	3 (25)	1.00c	
Any Infection (- giardia and Entamoeba)	90 (49)	13 (50)	30	6 (30)	61 (27)	0.79c		4 (7)	30 (16)	0.08b		4 (24)	63 (28)	1.00b		34 (15)	0 (0)	0.25b	

a = t-test b = Fisher's exact test c = Chi-square test d = Kruskal-Wallis test

Supplement 3.

PARTICIPANT ID: ______________________ INFANT ANEMIA STUDY

NUTRITION QUESTIONNAIRE

BREASTFEEDING:

1. Was your child breast fed? ☐ Yes ☐ No

IF YES:

2. Duration of exclusive breastfeeding (months) ________________________________

3. In the first year of life, were you breastfeeding another child at the same time? ☐ Yes ☐ No

IF NO:

4. Did you give formula? (Enfamil, Similac, other?) ☐ Yes ☐ No

5. Did you give animal milk? (Cow, goat, sheep?) ☐ Yes ☐ No

FIRST FOODS (COMPLEMENTARY FOODS)

1. At what age were first food(s)/solid foods (age in months) introduced? ☐ 0-3 ☐ 3-6 ☐ 6-9 ☐ 9+

2. What was the first food you introduced? ____

3. All foods introduced by one year of age? ☐ Yes ☐ No

FOOD SECURITY: Now I would like to ask you about your food consumption in the last 12 months. During the last 12 months, was there a time when:

1. You were worried you would run out of food because of lack of money or other resources? ☐ Yes ☐ No

2. You were unable to eat health and nutritious food because of a lack of money or other resources? ☐ Yes ☐ No

3. You ate only a few kinds of foods because of a lack of money or other resources? ☐ Yes ☐ No

4. You had to skip a meal because there was not enough money or other resources to get food? ☐ Yes ☐ No

5. You ate less than you thought you should because of a lack of money or other resources? ☐ Yes ☐ No

6. Your household ran out of food because of a lack of money or other resources? ☐ Yes ☐ No

7. You were hungry but did not eat because there was not enough money or other resources for food? ☐ Yes ☐ No

8. You went without eating for a whole day because of a lack of money or other resources? ☐ Yes ☐ No

9. Was there a time when your child did not eat healthy and nutritious foods because of a lack of money or other resources? ☐ Yes ☐ No

10. Was there a time when your child(ren) was not given enough food because of a lack of money or other resources? ☐ Yes ☐ No

11. Do you ever have to make smaller portions for meals? ☐ Yes ☐ No

12. Is it harder to have regular meals/foods at certain times of year? ☐ Yes ☐ No

If yes, when? ____

13. How many times a day does your child eat? ____

OTHER DIETARY PRACTICES: Now I'm going to ask you about other dietary practices you and your infant may have

1. Did you chew slate during pregnancy? ☐ Yes

☐ No

If yes, how many times/week? ____

2. Does your child eat non-edible foods (dirt, slate, etc?) ☐ Yes ☐ No

If yes, how many times/week?

____

DIETARY DIVERSITY: Please describe whether you and your child(ren) ate the foods listed and how many times you the foods during the day and night in the past week, whether at home or outside the home.

	FOOD GROUP	EXAMPLES	MOTHER			INFANT		
			YES/ NO	#TIME S/DAY	#TIMES/ WEEK	YES/ NO	#TIMES/ DAY	#TIMES/ WEEK
1	BREADS/CERE ALS	Ugali, Chapati, Githeri, Rice, Corn/Maize, Wheat, Millet, Other grains, Porridge						
2	VITAMIN A RICH VEGETABLES AND TUBERS	Sweet potatoes, Pumpkin, Carrots, Squash, Red sweet pepper						
3	WHITE TUBERS AND ROOTS	White potatoes, White Yams, White cassava						
4	DARK LEAFY VEGETABLES	Sukuma wiki, Mchicha (Amaranth), Cassava leaves, Spinach						
5	OTHER VEGETABLES	Tomato, Onion, Eggplant, Cabbage, etc.						
6	VITAMIN A RICH FRUITS	Ripe Mangoes, Cantaloups, Pawpaw (Papaya), Apricots, Pineapple						
7	OTHER FRUITS	Watermelon, Pineapple, Wild fruits, Bananas (raw or cooked), etc.						
8	ORGAN MEAT (IRON RICH)	Liver, Kidney, Heart or Other organ meats						
9	FLESH MEATS	Beef (Nyama), Goat, Lamb, Rabbit, Chicken, Duck, Wild Game, Other meat						
10	EGGS	Chicken, Duck, Guinea hen or						

		Other						
11	FISH	Omena-silver cypinid, Rastrineobola argentea, Papa-shark, Shellfish, Tilapia, King fish, Dried fish						
12	LEGUMES, NUTS, SEEDS	Githeri, Beans, Cowpeas, Lentils, Nuts or foods made from these						
13	MILK/MILK PRODUCTS	Milk, Cheese, Yogurt or other milk products						
14	OILS AND FATS	Oils, Fats, Butter						
15	RED PALM PRODUCTS	Palm nut or pulp sauce						
16	SWEETS	Sugar, Honey, Chocolates, Candies, Cookies, Case						
17	BEVERAGES/CONDIMENTS	Tea, Coffee, Sugary drinks (juice)						
18	OTHER	Nazi (coconut)						

SICKLE DISEASE/TRAIT:

1. Has your child been diagnosed with sickle disease or trait? ☐ Yes ☐ No
2. Has anyone in your child's immediate family (parent, siblings) been diagnosed with sickle disease or trait? ☐ Yes ☐ No

EXAMINER INITIALS: ___________________________ DATE: __________________

Supplement 4.

A.

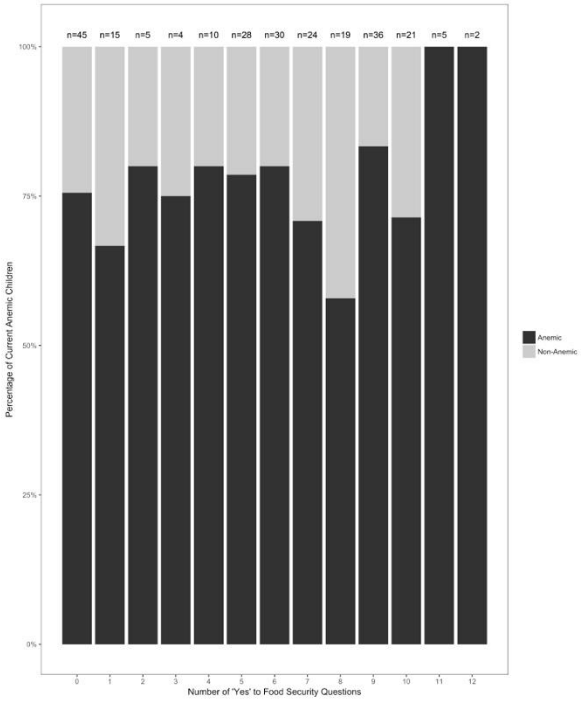

B.

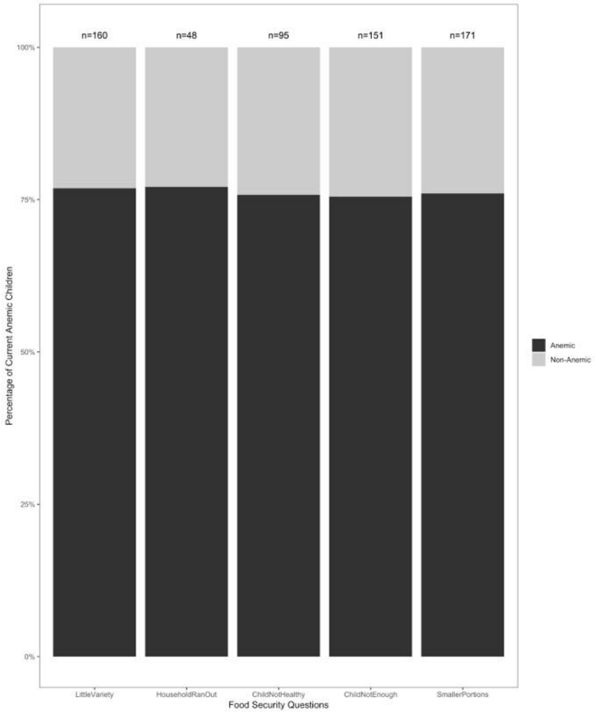

Supplement 5.

**Supplement 5: Model Estimates**		
Variables	Adjusted for age and gender	
	β estimate (95% CI)	p-value
Model for Infant Hb at different ages		
Maternal Malaria		
6 month Hb (n=198)	0.24 (-0.34, 0.82)	0.42
12 month Hb (n=204)	-0.35 (-0.87, 0.16)	0.17
18 month Hb (n=159)	-0.19 (-0.83, 0.46)	0.57
24 month Hb (n=104)	-0.60 (-1.37, 0.17)	0.13
30 month Hb (n=44)	-0.89 (-2.13, 0.36)	0.16
36 month Hb (n=8, none had maternal malaria)	NA	NA
Current Hb (n=210)	-0.52 (-1.04, -0.01)	0.04
